# Histological and Histomorphometric Comparison of Innovative Dental Implants Laser Obtained: Animal Pilot Study

**DOI:** 10.3390/ma14081830

**Published:** 2021-04-07

**Authors:** Mastrangelo Filiberto, Botticelli Daniele, Bengazi Franco, Scarano Antonio, Piattelli Adriano, Iezzi Giovanna, Quaresima Raimondo

**Affiliations:** 1 Clinical and Experimental Medicine Department, School of Dentistry, University of Foggia, 77100 Foggia, Italy; 2 ARDEC Academy, 47923 Rimini, Italy; daniele.botticelli@gmail.com (B.D.); bengazi@tin.it (B.F.); 3 Faculty of Dentistry, University of Medical Science, La Habana 10400, Cuba; 4 Department of Medical, Oral and Biotechnological Sciences, School of Dentistry, University of Chieti, 66100 Chieti, Italy; ascarano@unich.it (S.A.); apiattelli@unich.it (P.A.); giovanna.iezzi@unich.it (I.G.); 5 Department of Civil, Architecture and Environmental Engineering, University of L’Aquila, 67100 L’Aquila, Italy; raimondo.quaresima@univaq.it

**Keywords:** laser treatment, dental implants, sandblasted and acid etching implant, sheep animal model, histological and histomorphometrical analysis, bone to implant contact (BIC), dynamic osseointegration index

## Abstract

Objective: Evaluation of the in vivo bone response of two innovative titanium surfaces ytterbium laser active fiber obtained (L1-L2) compared to a sandblasted and acid etched (SBAE) during early phase of osseointegration. Material and Methods: Three implant groups with the same macroscopic features were obtained (L1-L2-SBAE) to promote specific surface characteristics. Scanning electron microscopy, profilometric evaluation, X-ray spectrometry, and diffraction analysis were performed. For each group, six implants were placed in the tibiae of three Peli Buey sheep, and histologic, histomorphometric analysis, bone to implant contact (BIC), and the Dynamic Osseointegration index (DOI) were performed. Results: During the early phases of osseointegration, the histological and histomorphometric results showed significant differences between L1-L2-SBAE implants. At 15 and 30 days, histological analysis detected a newly bone formation around all specimens with an higher vital bone in L2 compared to L1 and SBAE both in cortical and in poor-quality marrow bone. At same time, histomorphometric analysis showed significantly higher BIC values in L2 (42.1 ± 2.6 and 82.4 ± 2.2) compared to L1 (5.2 ± 3.1 and 56.2 ± 1.3) and SBAE (23.3 ± 3.9 and 77.3 ± 0.4). DOI medium value showed a higher rate in L2 (2.83) compared to SBAE (2.60) and L1 (1.91). Conclusions: With the limitations of this pilot study, it is possible to assess that the titanium surface characteristics, and not the technologies used to obtain the modification, played a crucial role during the osseointegration process. Histological, histomorphometric, BIC, and DOI evaluation showed a significantly higher rate in L2 specimens compared to others, confirming that the implant surface could increase the bone response in cortical or marrow poor quality bone during the initial phases of osseointegration.

## 1. Introduction

In modern dentistry, osseointegrated implants represent the standard treatment to partially and completely edentulous patients’ prosthetic rehabilitations [1]. In 1983, Brånemark defined osseointegration as a biological process with a structural and functional direct contact between the vital bone and implant fixture [2,3]. Primary and secondary stability of the fixture promoted the osseointegration and were related with several factors including bone density, implant shape, macrodesign, and surface characteristics [4,5]. During healing time, the bone formation around the implant is produced by different biological events, with molecular and cellular activations, started after implant placement [6,7,8]. Several studies showed implant surface as a crucial factor involved in the implant osseointegration through the clot adhesion, cell migration, increasing the matrix deposition, and to promote the bone to implant contact (BIC) [9,10,11,12]. Traditional or innovative titanium surface treatments, as machined, chemical, electrochemical, or laser, showed relevant osseointegration process differences [5,13,14,15,16,17,18,19] promoting cell alignments and migration [20,21]. Using different energy sources, powers, and times, the laser technology could be able to design macro, micro, and nano textured surfaces characterized by peaks, grooves, and pits that are constantly reproducible [18,20,22]. Important novel laser treatments produced a large palette of colors with interesting optical and physical features for aesthetic dental implant solution [20,23]. Currently, a rehabilitation time reduction, a predictable long-term success, and better aesthetic results are required both by the patients and the clinicians [24]. Osseointegration is not a static biological process, but dynamic, that evolves and changes throughout life [20,25]. To better understand this process, in addition with the bone to implant contact percentage (BIC), the authors proposed the Dynamic Osseointegration Index (DOI).

In the present study, the different behavior between two different innovative titanium surfaces that were laser modified, compared with a sandblasted and acid etched implant used as a control, was evaluated with histologic and histomorphometric analysis, BIC, and Dynamic Osseointegration Index.

## 2. Material and Methods

### 2.1. Surface Physical Analysis

Same Titanium Grade 5 implants (Ti6Al4V) of 3.3 mm in diameter and 8.5 mm in height were used to compare bone responses and prevent errors or bias. Type and dimensions of the implant fixture were selected according to sheep tibia surgical site dimension. The implants’ physical characteristics were analyzed by Scanning Electron Microscopy (SEM) (XL30CP—Philips, Eindhoven, The Netherlands). The surface characteristics were evaluated with contact portable profilometer (Taylor Hobson, Subsonic 3+, Leicester, UK) and the absolute values of the profile heights over the evaluation length (Ra), the root mean square average (Rq), the vertical distance from the highest peak to the lowest valley (Rz also known as Rtm values), the maximum peak height of the profile (Ry), and the mean spacing between peaks (Sm also known as RSm) were performed according to ISO 4287:1997. Linear measurements of 8 mm (points density of 500/mm) along the external fixture profile were evaluated in all samples in triplicate. For the profilometer, manufacture specification reports a cut-off of 0.25, 0.80, and 0.10 mm, 2RC and phase correct Gaussian filters, and a transmission of the selected cut-off to 75%. A normal probability test (*t*-test) was used to evaluate the statistical significance of the normal distribution of the surface roughness parameter values. The average of 10 analyzed samples were recorded in triplicate and *p* ≤ 0.005 difference between the samples was considered statistically significant.

### 2.2. Surface Chemical Analysis

At 100× magnification for 1 min by 20 kV energy dispersive spectroscopy (EDS—Oxford, Inca Energy 250, High Wycombe, UK), the chemical surface analysis was assessed. Moreover, with X-ray diffraction analysis (XRD—Philips PW 172, 9 AE Eindhoven, The Netherlands) using CuKα radiation and operating at 30 kV/40 mA with a 1st divergence slit with a step size of 0.02° 2θ in the 2θ range of 5–90°, implants were analyzed to verify the formation of harmful compounds. Crystalline phases were evaluate using the Joint Committee of Powder Diffraction Standard data base (JPDS) of the International Centre for Diffraction Chemical Data [26].

### 2.3. Specimens Preparation

The Ethics Committee for Animal Research of the Veterinary School of the University of La Havana (Havana, Cuba) approved the study protocol, which followed guidelines established by the Comitè de Etica de la Investigation de la Facultà de Estomatologia de l’Universidad de Ciencias Medicas de La Habana—Cuba with protocol 05/2018 titled: “A new laser treated implant surface: experimental pilot study in the tibia of the sheep”. Eighteen titanium implants (Premium-Sweden & Martina, Due Carrare—Padova, Italy) were used and selected in 3 groups. In a clean room, implants were ablated with ytterbium laser active fiber in two completely different conditions to obtain Laser 1 (L1) and Laser 2 (L2) surfaces. After treatments, all samples were ultrasonically cleaned, with ethanol and demineralized water, to remove contaminants, dried in an oven, and sterilized. Group sand blasted and acid etched (SBAE) dental implants were provided by Sweden & Martina (Due Carraie—Padova, Italy) and obtained with sand blasted and acid etched according to ZirTi specific registered procedure for Premium Implants (Sweden & Martina, Due Carrare—Padova, Italy).

### 2.4. In Vivo Surgery

**Figure 1:**
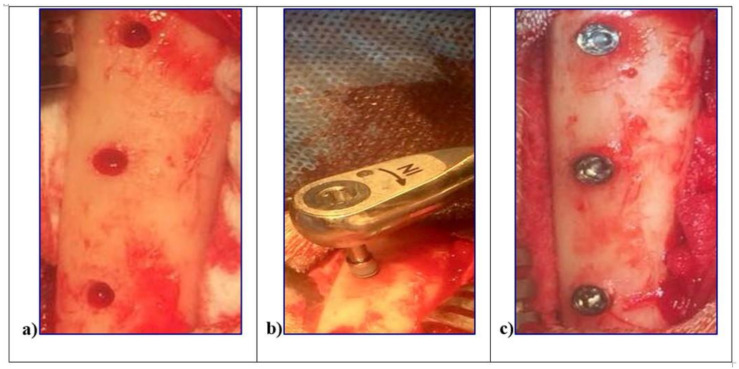
In vivo surgical procedures of the L1, L2, and sandblasted and acid etched (SBAE) implants insertion in sheep tibiae. (**a**) Surgical sites preparation. (**b**) Implant fixture insertion at 8.5 mm depth from the outer contour of the tibia. (**c**) Simultaneously, implants insertion to the top of the cortical bony crest.

The surgery was performed in accordance with the Code of Good Practice of the Laboratory of the CETEX (Centro de Toxicologıa Experimental, La Habana, Cuba) and the Code for the use of laboratory animals of the CENPALAB (Centro Nacional para la Produccion de Animales de Laboratorio, La Habana, Cuba) recommendations. All procedures were performed under anesthesia and veterinaries’ control to minimize the animals suffering. Three female “Peli-Buey” sheep, in good general health, mean weight 35 kg, and 3 years average age, were enrolled. Pregnancy or systemic disease, active infection or severe inflammation were indicated as exclusion criteria. At each surgical session, the animals were pre-anesthetized with atropine 0.02 mg/kg i.v. (Mayne Pharma, Napoli, Italy) and anesthetized i.m. with 0.04 mg/kg metedomidine (Medetor^®®^—Virbac, Glattbrugg, Switzerland) added to 5 mg/kg of ketamine-50 (Liorad, Havana, Cuba) after premedication. The anesthesia was maintained with 2–3% Isoflurane (Vet^®®^—Merial, Tolosa, France) with O_2_ at 95%. Local anesthesia was also provide with Mepivacaine (Mayne Pharma, Napoli, Italy). The blood pressure as well as the O_2_ perfusion was constantly monitored. The legs of the sheep were shaved, disinfected with chlorhexidine di-gluconate, and incised in the facial aspect. The skin as well as the muscular fascia will be elevated, and the tibia bone plate denuded. Three surgical sites were obtained in each tibia using drill preparation with water-cooling, through the cortical bone layer and into the marrow compartment, up to a depth of 8.5 mm from the outer contour of the tibia. In each site, one implant L1, L2, and SBAE, with cover screws, was placed at the top of the cortical bony crest. To minimize potential errors and distinguish each implant typology, the bone bordering was differently marked with a bone screw for the future identification. Wounds were sutured with reabsorbable suture (Figure 1).

In order to obtain two periods of healing, all the animals were operated on in different surgical periods of 15 and 30 days. During the post-operative period Tramadol^®®^ (Altadol; Formevet, Milan, Italy) 2 mg/kg was administered twice a day for 5 days, and in the healing period, the animals were kept in sheepfolds with free access to water and food. Daily visits were performed to evaluate the clinical signs of healing and surgical complications. At the end of the experimental period, the animals were first anesthetized with 0.04 mg/kg metedomidine (Medetor^®®^—Virbac, Glattbrugg, Switzerland) added to 5 mg/kg of ketamine-50 (Liorad, Havana, Cuba), and then euthanized with 25 mEq of potassium chloride intravenously (Aica, La Habana, Cuba).

### 2.5. Histological Preparation

The tibia specimens were retrieved, trimmed, and immersed in 4% formaldehyde solution. The histological samples were performed in the laboratory of Histology at the Faculty of Odontology of the University of La Habana, Cuba. Block sections, each containing one implant, were dehydrated in a series of graded ethanol, and subsequently embedded in resin—Technovit 7200 (VLC Kulzer, Friedrichsdorf, Germany). Concerning the fixtures position in the tibiae, a precision transversal slice cuts (150 ± 200 μm) were carried out (Exakt1, Apparatebau, Norderstedt, Germany) along the centerline of the implants. The slices, very fine and extra fine grinded (silicon carbide P280, P320, P400, P600) (ISO/FEPA Grit designation), polished (diamonds suspension polycrystalline 9–3–1 μm), reduced to a thickness of about 60 μm (Exakt1, Apparatebau, Norderstedt, Germany-ATM QNESS, GMBH, 57636 Mammelzen, Germany), and stained with Stevenel’s blue and alizarin red.

### 2.6. Histologic and Histomorphometric Analysis

**Figure 2:**
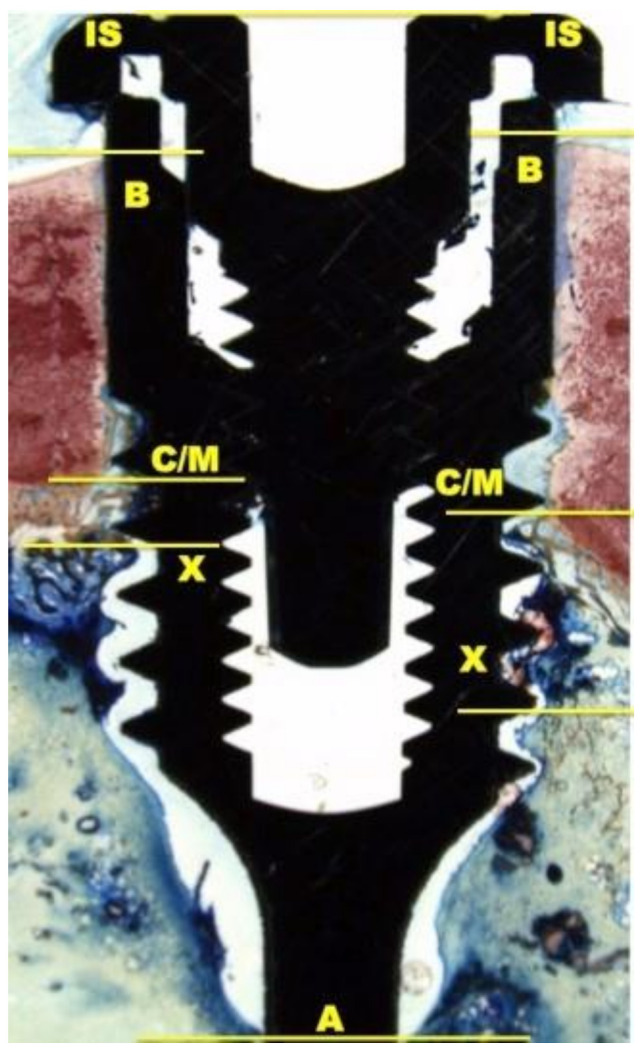
At 100× magnification, landmarks scheme adopted for the histological measurements: Shoulder of the implant (IS), the most coronal bone-to-implant contact point (B), the intersection between cortical and marrow bone compartments (C/M), the most apical bone-to-implant contact point (X), and the apex of the implant (A).

The histological and histomorphometrical analysis were performed after 15 and 30 days using a light microscope (Eclipse Ci—Nikon Corporation, Tokyo, Japan), digital video camera equipped (Digital Sight DS-2Mv; Nikon Corporation) connected to NIS-Elements D4.10 software (NIS.ai, Laboratory Imaging; Nikon Corporation, Melville, NY, USA). A high-resolution video camera was interfaced with a monitor and PC (Intel Pentium III 1200 MMX, Intel, Santa Clara, CA, USA). The image analysis was performed by digitizing pad (Matrix Vision GmbH, Oppenweiler, Germany) and a histomorphometry software (Image-Pro Plus 4.5, Media Cybernetics Inc., Immagini & Computer Snc, Milano, Italy). BIC was evaluated as the ratio between the linear surface of the implants firmly in contact with the mineralized bone matrix compared with the total implant surface. In particular, at 100× magnification, the samples were compared with specific landmarks to identified the shoulder of the implant (IS), the most coronal bone-to-implant contact point (B), the intersection between cortical and marrow bone compartments (C/M), the most apical bone-to-implant contact point (X), and the apex of the implant (A) and parallel linear distance to the implant long axis were detected (Figure 2). Moreover, on both sides of the implant, the percentages of new and old bone, yellow bone marrow, and residual tissue (clot and bone debris/particles), in contact with the implant surface, were performed between B and C/M, C/M and A, and between B and A (Figure 2). Using Adobe Photoshop software (Adobe Photoshop & Premier Elements 2020, Adobe, San Jose, CA, USA), the different hues of the bone, representative of old or new bone stages, were detected according to International Commission on Illumination (CIE) in the color space L*, a*, b* (CIELAB). D50 2° were chosen as illuminant and reference angle for input values.

### 2.7. Dynamic Osseointegration Index

The quality and value of osseointegration are crucial factors of a dental implant success. Nowadays, starting from histomorphometric analysis, bone to implant contact was adopted to measure the osseointegration rates and bone maturation, but only at the end of the healing time. It should be necessary to achieve values during all healing time, especially during the early phases of osseointegration. Therefore, the authors proposed, in addition, a dynamic osseointegration index (DOI), using the BIC data, to obtain more complementary information of bone behavior and different contact osseointegration values during all healing times. The DOI in L1, L2, and SBAE specimens was evaluated around the implant fixture at 15 and 30 days to understand if the surfaces are able to influence the bone behavior during the early phases of osseointegration. The DOI was expressed as ratio between the BIC percentage and the numbers of days. In particular, the medium Dynamic Osseointegration Index of the three different implant groups was the mean value of BIC percentage divided for 30 days, while DOI_1_ was the BIC percentage at 15 days divided by first the fifteen days, and the DOI_2_ was the BIC value at 30 days divided by the second fifteen days.

### 2.8. Histomorphometric Statistical Analysis

Mean values and standard deviations were calculated for each outcome variable. At 15 and 30 days, differences between L1, L2, and SBAE implants were evaluated with Wilcoxon test included in the SPSS Statistics 19 (IBM Inc., Chicago, IL, USA). The level of significance was set at a *p* ≤ 0.05.

## 3. Results

**Figure 3:**
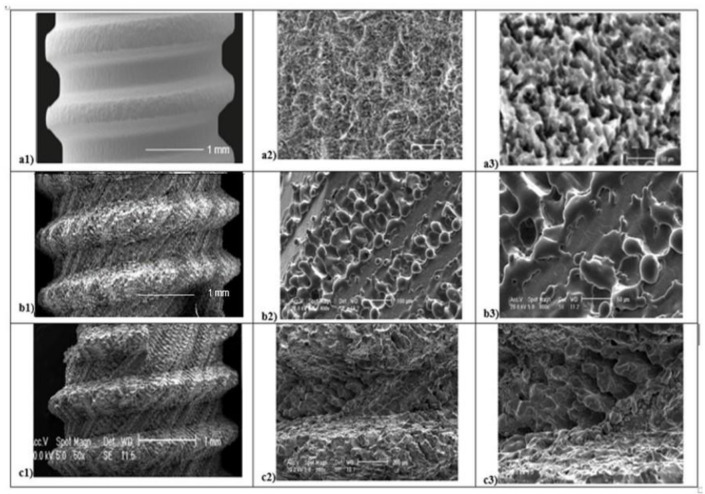
Scanning Electron Microscope (SEM) analysis of titanium surfaces at 50×, 400×, and 800×: SBAE (**a1**–**a2**–**a3**), L1 (**b1**–**b2**–**b3**), and L2 (**c1**–**c2**–**c3**) (black marked scale bars reported are equal for each set of magnifications).

Several micro and macroscopic topography surface differences were detected in all 3 groups analyzed (Figure 3). At low magnification (50×), SEM evaluation and SBAE samples showed a fine surface structure with regular roughness. At high magnification (400×), irregular micro-roughness with fine sharp crests alternated to different deep valleys without a reproducible texture in various areas of SBAE surface were performed (Figure 3a1). At higher magnification (800×), it is possible to observe a very low diffuse porosity within 15 and 25 µm at the same depth (Figure 3a2,a3). At low magnification, the L1 sample showed a specific fine diagonal bands micro topography (Figure 3b1). At higher magnification (400×–800×), it is possible to observe the presence of two roughened bands (respectively, 200 and 100 µm width spaced of 20 µm) that alternate by a smooth band of 50 µm width (Figure 3b2,b3). The porosity within 15 and 50 µm is located in the ablated areas. At low magnification (50×), L2 samples showed a marked macroscopic porosity consisting of only diagonal parallel highly grooved bands (Figure 3c1). At 400× magnification, an uniform series of micro-structures formed by narrow ridge structures and diffuse different valleys with various deep depressions were observed (Figure 3c2). At higher magnification (800×), L2 showed a constant nano-crests alternation of nano-projection structure villi-like interspaced between diffuse valleys with different nano-depth, obtained through a complex laser titanium treatment (Figure 3c3). Porosity is homogenously distribute and ranged within 5 and 50 µm.

**Figure 4:**
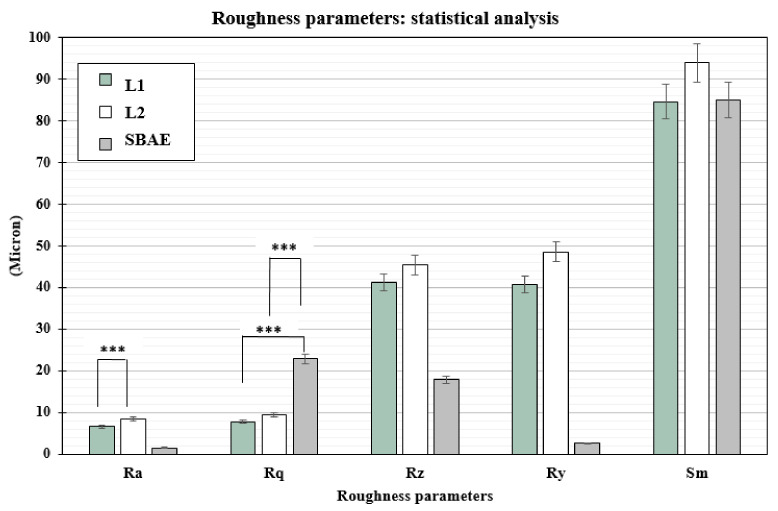
*t*-test statistically significance relationship between the profilometric data recorded: *p*-values ≤ 0.005 was considered positive. Ra of L1 vs. L2 (0.0013), and Rq of L1 vs. SBAE (0.0001) and L1 vs. SBAE (0.0001), showed surface textures statistically significant differences (*** *p* ≤ 0.005).

The profilometric evaluation of the 3 groups showed Ra, Rq, Rz, Ry, and Sm different surface roughness values (Table 1). The SBAE average linear surface roughness (Ra) detected 1.51 µm (±0.18) value, very different from L1 (6.58 µm ± 0.72) and L2 samples (8.51 µm ± 0.84). The root-mean-square (Rq) value provided lower value in L1 (7.87 µm ± 0.91) than L2 (9.42 µm ± 0.94) and SBAE (22.87 µm ± 1.30). The average distance between the highest peak and lowest valley (Rz), higher in L2 (45.35 µm ± 9.35) than L1 (41.16 ± 8.25 µm) and SBAE (17.90 ± 1.37 µm), signaling a more complex and irregular surface texture. The laser treatment surface maximum height of the profile (Ry) showed closed value 40.73 µm (±9.92) in L1 and 48.60 µm (±9.92) in L2, respectively, compared with the lower value 02.62 µm (±0.12) in SBAE. The mean spacing between peaks (Sm) found similar values within L1 (84.70 µm ± 7.92) and SBAE (85.00 µm ± 8.63) compared to the higher value of L2 (96.33 µm ± 10.69). The Ra of L1 vs. L2 (0.0013) and the Rq of L1 vs. SBAE (0.0001) and of L2 vs. SBAE (0.0001) showed surface textures statistically significant differences (*p* ≤ 0.005) (Figure 4). The surface textures showed average higher value differences between L1–L2–SBAE. All sample were evaluated in triplicate.

### 3.1. Surface Chemical Analysis

**Figure 5:**
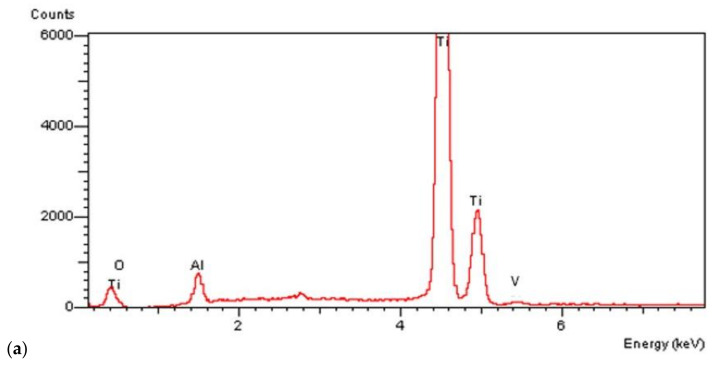

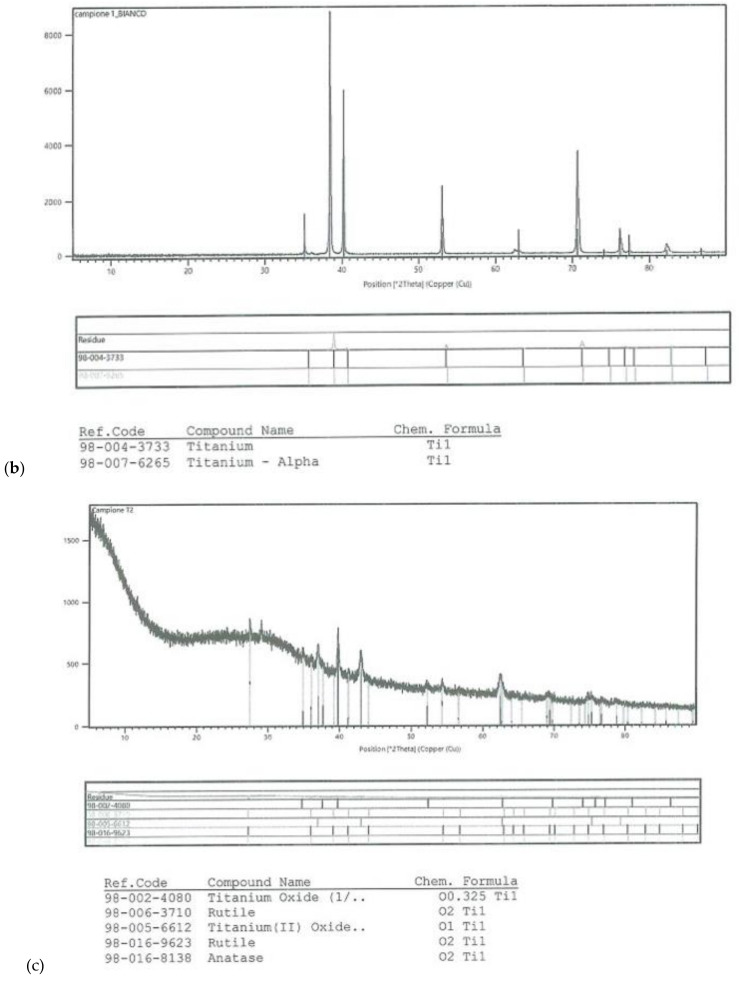

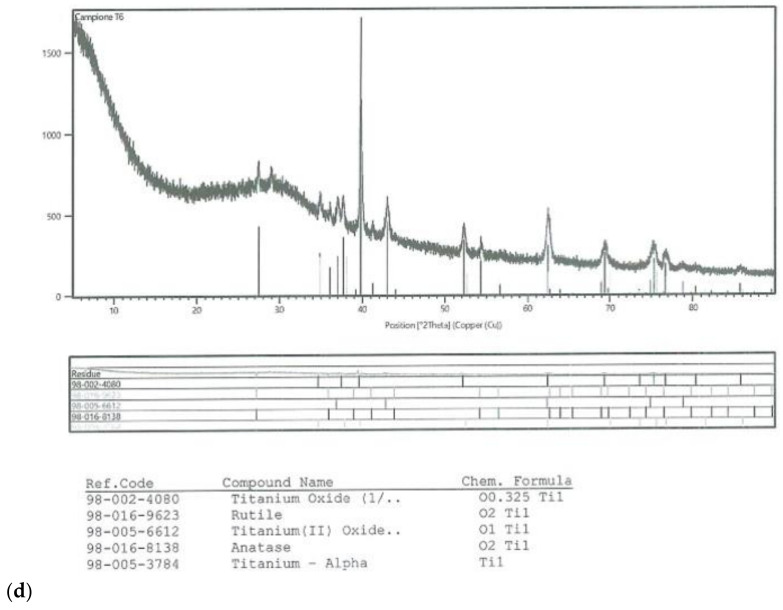
Energy Dispersive X-ray (EDS) analysis of L1, L2, and SBAE (**a**) showed same values of Ti, Al, and V. X-ray power diffraction analysis of SBAE (**b**), L1 (**c**), and L2 (**d**) showed the absence of non-biocompatible compounds.

On all the titanium surfaces analyzed, Energy Dispersive X-ray (EDS) and X-ray Power Diffraction (XRD) analyses showed the presence of biocompatible elements (Oxygen, Titanium, Aluminum, and Vanadium and compounds) (Figure 5a–d).

### 3.2. Histological Analysis

**Figure 6:**
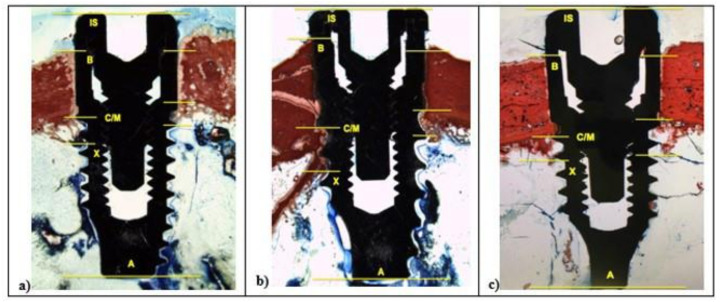
After 15 days, histological analysis of SBAE (**a**), L1 (**b**), and L2 (**c**) of the osseointegration healing process at 100× magnification.

After two weeks, in all samples around implant surface, few new bone (CM-X) was observed both in cortical, then marrow bone compartments with different color intensity. At 100× magnification, low red color intensity was detected around the implant fixture compared to the higher color intensity of the old cortical bone. All fixtures showed peri-implant bone reabsorption coupled with bone formation processes and a large area of bone marrow. In all samples, IS/B area was present with a higher space in L1 and SBAE compared to L2 (Figure 6).

**Figure 7:**
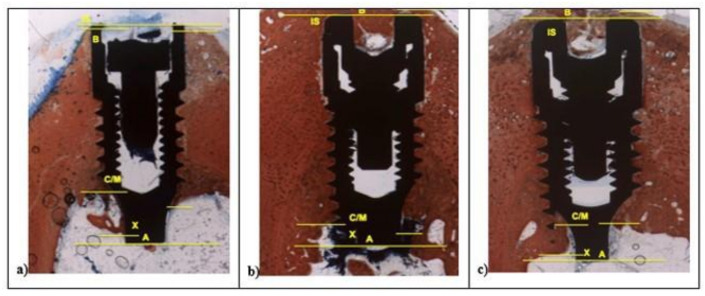
After 30 days, histological analysis of SBAE (**a**), L1 (**b**), and L2 (**c**) of the osseointegration healing process (100× magnification).

In L1 and SBAE samples, the cortical bone area showed lower red intensity around the crestal fixture (Figure 6c) (L* = +42.75, a* = +19.37, b* = +26.27), while in L2 a higher red color intensity was detected (L* = +29.12, a* = +23.14, b* = +19.26), suggesting a clear difference of the bone remodeling process between the 3 different surfaces (Figure 6b). The L1 sample showed CM-X mean value 0.94 mm (±0.19), L2 4.81 mm (±0.28), and SBAE 2.63 mm (±0.13) (Figure 6a). After 30 days, in all samples a marrow space reduction and a high contact of the bone with the implant was detected (Figure 7).

**Figure 8:**
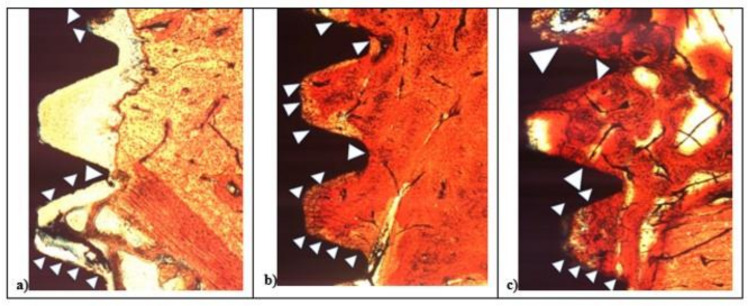
After 15 days histological analysis of SBAE (**a**), L1 (**b**), and L2 (**c**) of the osseointegration healing process at 200× magnification.

In the L1 fixture, low red color intensity of the bone around the crestalarea (L* = +47.70, a* = +20.76, b* = +26.48) and little IS/B distance was detected. Homogeneous bone layer completely covered the implant body. In L1, the apical areas showed spot bone deposition, and different areas were without bone to implant contact (Figure 7a), while L2 and SBAE samples were completely covered with a thick layer of new bone (Figure 7b,c). A higher red color intensity around the crestal area in L2 was detected compared to the SBAE. However, in L2 it was possible to observe little spot areas of peri-implant new bone deposition with low red color intensity (L* = +42.54, a* = +17.36, b* = +24.53) (Figure 7b). The body of L2 and SBAE fixture was totally covered by the bone with a deep red color intensity. An intimate contact to the implant body was observed in L2 sample (Figure 7b). In SBAE samples, the bone in contact to the implant body showed a lower red color intensity (Figure 7c). At 200× magnification, clear bone difference behavior between the three surfaces was detected (Figure 8).

In L1, the bone apposition was not continuous, and several areas of the implant surface showed no bone contact. In the deeper zone between implant threads, the bone contact was present only in a few spots (Figure 8a). In the L2 and SBAE, the bone apposition was more continuous, showing the bone flowing above the implant surfaces with small but significant differences (Figure 8b,c). In L2 sample, a homogeneous bone layer completely covered the implant surface in deep connection. The bone showed no color difference between the contact bone to the mature peri-implant lamellar bone, except in some lighter red spot areas (Figure 8b). In the SBAE sample, the bone closest to the implant showed several immature bone areas connected to the mature peri-implant lamellar bone and some areas totally without bone connection to the implant surface (Figure 8c).

### 3.3. Histomorphometric Analysis

The bone to implant contact around the implant showed a careful difference between the three groups analyzed (Table 2).

**Figure 9:**
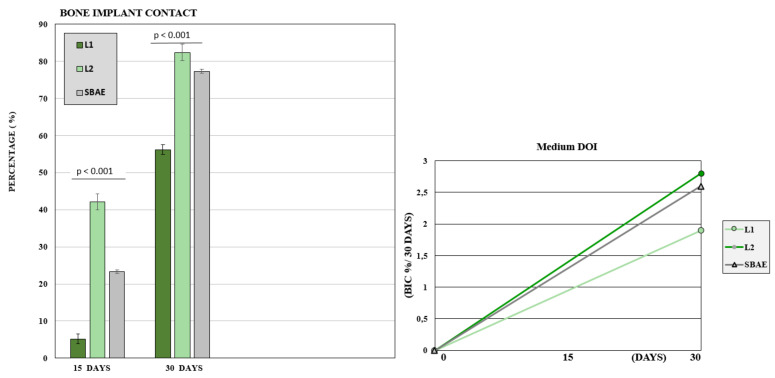
Bone to Implant Contact of L1, L2, and SBAE surfaces histomorphometric analysis. At 15 and 30 days, the L2 samples showed a statistically significantly high BIC percentage compared to SBAE and L1 (*p* < 0.05). Dynamic Osseointegration Index: Medium value after 30 days. The higher value is obtained for L2 compared to L1 and SBAE.

After 15 days, the BIC value was low with 23 (±3.8) mean rate. The L1 sample showed a mean BIC value of 5.2 (±3.1), L2 value of 42.1 (±2.6), and SBAE value of 23.3 (±3.9). After 30 days, a great bone improvement around the implant fixture was detected, but significant differences between the three groups. In L1, the mean BIC value was 56.2 (±1.3), in L2 was 82.4 (±2.2), and SBAE was 77.3 (±0.4). The Wilcoxon test statistical analysis, showed a statistically significant difference of L2 BIC percentage compared to SBAE or L1 (*p* < 0.05) in both periods analyzed (15 or 30 days) (Figure 9). The Dynamic Osseointegration Index medium value showed the higher result in L2 samples (2.83), high in SBAE (2.60), while in L1 the value was lower (1.91). After 15 days, DOI_1_ in L2 samples showed higher value (2.81) compared to SBAE (1.5) and L1 (0.38). After 30 days, DOI_2_ in SBAE samples was higher (3.6) compared with L1 (3.4), and L2 (2.83) samples (Figure 9).

## 4. Discussion

Dental implantology is a standard procedure for edentulism rehabilitation with high predictability rates [27,28,29,30]. Several studies have been carried out to demonstrate the osseointegration and its key factors [31,32,33,34]. However, in several studies, there are different bias and errors that do not allow us to understand the titanium surface role in osseointegration process because often only in vitro experimental studies are performed with oncological cells [9,35] able to grow on all surfaces, but above all these model are unable to reproduce the proper biological osseointegration process. Furthermore, in several studies, the new surfaces are compared with machined surfaces as a control [14,36,37] and again, quantitative morphological and non-qualitative biological analyzes are performed to determine if a surface influences the osseointegration [38,39].

Finally, several in vitro studies were performed without in vivo human or animal research on mesenchymal cells or osteoblasts [40,41,42].

The present study would understand if and how the implant surface plays a crucial role in the osseointegration, if the technology used to obtain the surface modification is able to influence the bone formation around the implant, and if the osseointegration is a constant biological process.

Currently, there is no agreement if the implant surface plays a crucial role during osseointegration and how it performs. Indeed, in 2008 and 2012 in animal model and in vitro study, respectively, Yeo et al. [43] and Jimbo at al. [44] showed how the design factor and the surface characteristics seem to not play a crucial role in the early bone response. In 2012, Choi at al. [45] in rabbit model and in 2013 Larsson et al. [46] in different surface-modified titanium implants with different characteristics detected no significant difference in the bone response two weeks after implant insertion. Instead, in 2016, Trisi et al. [14] affirmed that the uniform roughness of a laser treated implant surface showed significantly higher BIC percentage compared to machined implant surfaces in the sheep animal model. In 2009, Lang et al. [47] in an experimental study, and Wennerberg in a systematic review [11], affirmed that the surface properties seem to determine the osseointegration process. In 2010, Teté et al. in vitro showed how a novel anodized nano-titanium implant surface, compared with sandblasted and machined, influenced human osteoblasts behavior and growth [6]. In 2019, Romero-Ruiz et al. in minipig showed after 4–8 weeks a high osteoconductive activity and mature lamellar bone tissue production in SLA^®®^ and SLActive^®®^ compared to ContacTi^®®^ surfaces [48]. Szmukler-Moncler et al. in 2004 affirmed how 10–20 surface micron/macro-roughness were able to promote bone tissues growth inducing stable anchorage, while micro-roughness around 1–2 micron favors cell adhesion and proliferation on titanium surfaces [49]. In 2000, Ricci et al. [25], and in 2008, Weiner et al. [18], showed a closer bone and soft tissue adaptation induced by laser micro-textured surfaces in BioLok Laser-Lok^®®^ implant system. In previous in vitro research carried out and published in 2020, the authors showed the active role of the implant surface during the initial phases of bone matrix deposition and growth onto different surfaces compared with the sandblasted and acid-etched (SBAE) as a gold standard [20]. In the present in vivo study, to reduce the odds and to reproduce the natural bone healing conditions, the authors placed dental implants with the same height and diameter in sheep tibia. Two different ytterbium laser surfaces were textured with different complex procedures to obtain two different innovative tailor-made titanium implants compared with a Zirti sandblasted and acid etching surface used as control.

### 4.1. Histological Analysis

After 15 days, the histological analysis showed a clear difference of osseointegration process around the three different implants with a higher red color intensity and high CM-X mean value in L2 implant compared with L1 and SBAE. After 30 days, a high bone contact to the implant fixture was detected in all samples. However, at high magnification, L2 and SBAE samples were completely covered with a thick layer of new bone, while in L1, the apical areas showed only spot bone deposition area, and several sites showed no bone to implant contact.

### 4.2. Histomorphometric Analysis

The histomorphometric analysis verified the histological data at 15 and 30 days with significantly higher BIC value in L2 sample (42.1 ± 2.6 and 82.4 ± 2.2) compared with L1 and SBAE. The L1 BIC results showed no significant differences with Ivanoff [50] and Trisi [14] studies confirming lower osseointegration values compared to L2 and SBAE samples. Data obtained in animal model confirmed the surface active role during early phases of the osseointegration. The higher bone differences observed in L2 implants compared to L1 and SBAE showed how the micro and nano-textures play a crucial role during healing time with an improvement of bone formation related to the high value of the roughness nano-scale and the complexity of the titanium structure. The physical titanium surface complex texturing like to the dehydrate bone structure, seem to promote the bone adhesion and growth around the implants. Additionally, the results showed how the alone surface production technology is not able to influence the bone formation and the quality of the osseointegration. Indeed considering the two laser implants, during healing time, higher bone improvement and bone matrix deposition was observed in L2 compared to L1. Analyzing deeply the different results between L2 and SBAE implants, the surface texture morphology, and not the manufacturing technology process, seems to be able to influence the osseointegration, promoting in less time clot adhesion, cells migration, osteoblasts proliferation, and bone matrix deposition. Regarding the third question, the DOI index proposed by the authors in addition to the BIC percentage, confirmed the dynamic model of the osseointegration biological process. The DOI data highlights the different bone behavior around dental implants. The different growth rate showed higher average values in L2 (2.81) compared to SBAE (2.6) and L1 (1.8). After 15 days, L2 implants showed high DOI_1_ value (2.8) and abundant mature bone demonstrating a great surface affinity, compared to SBAE (1.5) and L1 (0.3). Onto these two surfaces, DOI seems to show a lower adhesion and proliferation capability of the bone around the implants. After further 15 days, the L1 (3.4) and SBAE (3.6) DOI_2_ showed increasing values, probably related to slow bone tissue recovery processes, compared to L2 implants (2.83) where the index remains steady stable, probably related to a bone matrix maturation. Furthermore, the statistical differences between L1, L2, and SBAE surfaces in Ra and Rq, in relation to the BIC and the DOI value reported, provide an interesting hypothesis that recognizes in the profile depth value of the titanium surface, the possible crucial factor able to positively influence the early phases of osseointegration process.

These promising preliminary data confirmed the different bone tissue behavior around dental implants and how the surface characteristics play a crucial role during early osseointegration phases. Moreover, the higher BIC and DOI_1_ values in the L2 implant are clinically relevant, in reducing the healing time and increasing the secondary stability allowing early or immediate implants loading rehabilitation.

## 5. Conclusions

Further medium-term studies are in progress, to confirm the results obtained in the present pilot study on a larger sample size for a more reliable statistical comparison. Nevertheless, with the limitations of this preliminary study, it is possible to assess that laser micro and nano-textured implant surfaces could guarantee high levels of osseointegration. However, the alone laser treatment, seems not to be able to obtain better osseointegration compared to gold standard implant surfaces. Therefore, the surface characteristics obtained with innovative ytterbium laser complex treatment, promoted a higher level of osseointegration with further developments in dentistry and maxillofacial surgical rehabilitations. Indeed, specific, reproducible, and complex texture laser characteristics, promoted high BIC and DOI values in L2 compared to L1 and SBAE implants. The adoption of the DOI index seems to be promising, in addition with the BIC index to evaluate the dynamic bone response to the implants surface during different phases of osseointegration.

## Figures and Tables

**Table 1:** The Ra, Rq, Rz, Ry, and Sm profilometric values of L1, L2, and SBAE titanium surfaces are reported in micron.

Sample	Ra	Rq	Rz	Ry	Sm
L1	06.58 (±0.72)	07.87 (±0.91)	41.16 (±8.25)	40.73(±9.92)	84.70 (±7.92)
L2	08.51 (±0.84)	09.42 (±0.94)	45.35 (±9.35)	48.60 (±9.92)	96.33 (±10.69)
SBAE	01.51 (±0.18)	22.87 (±1.30)	17.90 (±1.37)	02.62 (±0.12)	85.00 (±8.63)

**Table 2:** BIC% values recorded on the L1, L2, and SBAE titanium implants after 15 and 30 days.

Samples vs. Time	15 Days	30 Days
L1	5.2 ± 3.1	56.2 ± 1.3
L2	42.1 ± 2.6	82.4 ± 2.2
SBAE	23.3 ± 3.9	77.3 ± 0.4

## Data Availability

The data presented in this study are available on request from the corresponding author.
